# Messing up disorder: how do missense mutations in the tumor suppressor protein APC lead to cancer?

**DOI:** 10.1186/1476-4598-10-101

**Published:** 2011-08-22

**Authors:** David P Minde, Zeinab Anvarian, Stefan GD Rüdiger, Madelon M Maurice

**Affiliations:** 1 Cellular Protein Chemistry, Bijvoet Center for Biomolecular Research, Utrecht University, Padualaan 8, 3584CH Utrecht, The Netherlands; 2 Dept. of Cell Biology, University Medical Center Utrecht (UMCU), Rm G02.525, Heidelberglaan 100, 3584CX Utrecht, The Netherlands

## Abstract

**Summary**

Mutations in the adenomatous polyposis coli (*APC*) tumor suppressor gene strongly predispose to development of gastro-intestinal tumors. Central to the tumorigenic events in *APC *mutant cells is the uncontrolled stabilization and transcriptional activation of the protein β-catenin. Many questions remain as to how APC controls β-catenin degradation. Remarkably, the large C-terminal region of APC, which spans over 2000 amino acids and includes critical regions in downregulating β-catenin, is predicted to be natively unfolded. Here we discuss how this uncommonly large disordered region may help to coordinate the multiple cellular functions of APC. Recently, a significant number of germline and somatic missense mutations in the central region of APC were linked to tumorigenesis in the colon as well as extra-intestinal tissues. We classify and localize all currently known missense mutations in the APC structure. The molecular basis by which these mutations interfere with the function of APC remains unresolved. We propose several mechanisms by which cancer-related missense mutations in the large disordered domain of APC may interfere with tumor suppressor activity. Insight in the underlying molecular events will be invaluable in the development of novel strategies to counter dysregulated Wnt signaling by APC mutations in cancer.

## Introduction

Adenomatous polyposis coli (APC) is a key tumor suppressor gene that acts as a gatekeeper of intestinal epithelial homeostasis by restraining cytoplasmic cellular levels of β-catenin, the central activator of transcription in the Wnt signaling pathway. At the molecular level, APC co-scaffolds a multiprotein destruction complex, composed of the tumor suppressor Axin and the serine-threonine kinases GSK3β and CK1, which promotes the phosphorylation and subsequent ubiquitin-mediated degradation of β-catenin [1]. A Wnt-induced signal at the cell surface impedes the function of the APC-Axin complex, leading to the stabilization and nuclear import of β-catenin, followed by the formation of nuclear β-catenin/TCF complexes that activate target gene transcription [2,3]. Besides regulating proliferation and differentiation through Wnt/β-catenin signaling, APC controls multiple β-catenin-independent fundamental cellular processes. These include cell adhesion and migration, organization of the cytoskeleton, spindle formation and chromosome segregation [4,5]. The crucial role of APC in fundamental developmental cellular processes is illustrated by the embryonic lethality of homozygous *Apc*-knock-out mutations [6-8]. In this review, we focus on how the remarkable lack of structure in the large central domain of APC may facilitate its tumor suppressor function in the Wnt/β-catenin cascade. Furthermore, by classification and localization of known cancer-related APC missense mutations, we uncover different mutational spectra of germline and somatic missense mutations along the APC protein sequence, suggesting variation in functional relevance and mechanisms. We discuss how these missense mutations in the large unstructured region of APC may predispose to cancer.

### The large central domain of APC contains multiple domains that control Wnt signaling

**Figure 1:**
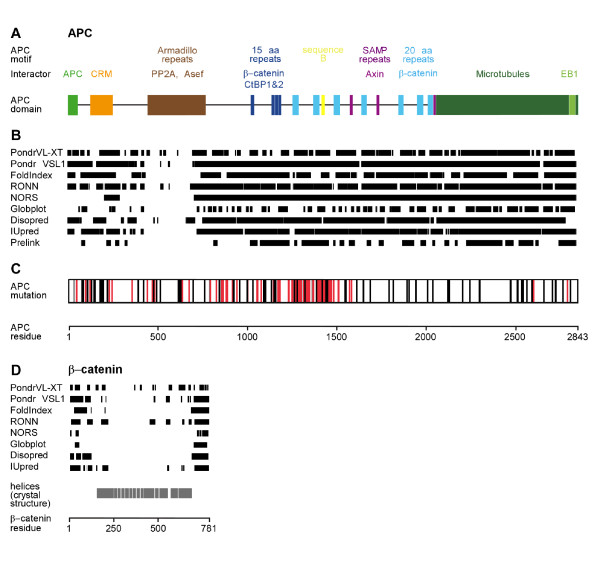
**The human APC protein carries a large predicted disordered domain which is frequently hit by missense mutations in cancer**. (A) Schematic representation of the APC scaffold protein and its protein interaction domains. Known interactors are APC (green), CRM1 (orange), PP2A (brown), β-catenin, CtBP (15aa repeats, blue), β-catenin (20 aa repeats, cyan), sequence B (yellow), Axin (SAMP-repeats, purple), Microtubules (dark green) and EB1 (light green). (B) Summary of disorder predictions performed for full-length human APC using different algorithms from publicly available servers [31-35]. Sequence segments with disorder probability above 50% are represented as black bars. (C) Distribution of missense mutations in the APC protein reported in various tumors, categorized as somatic (red), germline (black) or unknown origin (grey). Details on the location and nature of amino acid substitutions can be found in additional file 1, Table S1. (D) Summary of disorder predictions performed for β-catenin (black bars), done as in (B), and β-catenins's helical secondary structure elements as determined by crystallography (gray bars) [36].

APC is a 312 kDa protein composed of 2843 amino acid residues. It carries multiple designated segments with which it coordinates its multiple cellular functions (Figure 1A). The large central region of APC, spanning residues L1021-D2059, has been implicated in the downregulation of β-catenin [9,10]. It contains four 15aa repeat and seven 20aa repeat segments involved in β-catenin binding [11-13]. The 15aa repeat region also binds the transcriptional co-repressor CtBP1 and CtBP2, which prevents nuclear β-catenin activity and facilitates APC oligomerization and [14-16]. Interspersed with the 20aa β-catenin binding repeats three short recognition motifs, composed of the highly conserved LxECIxSAMP sequence (called SAMP motif), constitute binding sites for Axin [17-19]. The remarkably large number of β-catenin binding sites in the APC protein has instigated an area of intense research to search for the mechanistic role of the APC β-catenin binding repeats in the destruction complex.

Each 15aa repeat of APC binds to the structural groove formed by the armadillo repeats 5-10 on the surface of β-catenin in a phosphorylation-independent manner [13]. The 20aa repeats require phosphorylation of a consensus SXXSSLSXLS motif to convert into tight binding sites for β-catenin [20,21]. In addition, two negatively charged residues in the N-terminal flanking regions of the core 20aa repeats make significant contact with β-catenin by forming 2 salt bridges with K435 and K312 of β-catenin [20,21]. Once bound, one single phosphorylated 20aa repeat plus N-terminal flanking sequence of APC occupies almost the entire groove that spans from armadillo repeats 1 to 12 on the β-catenin surface. The bound conformation of one extended 20aa repeat is nearly identical to that of other functional binding partners of β-catenin, TCF [22] and E-cadherin [23], indicating that these proteins cannot bind β-catenin simultaneously.

Unlike their equal organization in binding motifs, individual 15aa and 20aa repeats in APC vary considerably in their binding affinities for β-catenin, with the tightest binding site being the phosphorylated third 20aa repeat (K_d _1.5 nM) [24]. Remarkably, the highly conserved second 20aa repeat completely lacks binding affinity for β-catenin, even in the phosphorylated state, likely due to the absence of some of the conserved residues in the N-terminal region flanking the core 20aa sequence. Phosphorylated APC competes with Axin for binding to β-catenin, whereas unphosphorylated does not [21,25]. Based on these findings, various models have been proposed on the mechanism by which phosphorylation of the 20aa repeats in APC may regulate β-catenin recruitment and turnover. In a first model, both the 15aa repeats of APC and the β-catenin binding domain in Axin bind β-catenin side by side to induce β-catenin phosphorylation by GSK3β. As soon as the third 20aa repeat of APC is phosphorylated it will bind phospho-β-catenin thereby releasing Axin from the complex [21,24]. This would facilitate the discharge and degradation of phospho-β-catenin and allow a new phosphorylation cycle to occur. In a second model, β-catenin first binds phosphorylated APC with high affinity. Subsequent dephosphorylation of APC is then required to weaken the interaction between APC and β-catenin, allowing transfer of β-catenin to Axin and phosphorylation by Axin-associated GSK3β [1]. This model is opposed by recent findings that demonstrate a crucial role of APC in protecting phosho-β-catenin from dephosphorylation by PP2A [26]. As a consequence, APC would stay tethered to phospho-β-catenin and directly deliver it to the E3 ligase β-TrCP for ubiquitination. In a third model, phosphorylation of APC accommodates the fluctuation in β-catenin levels in the cell in conditions of presence versus absence of a Wnt signal. During active Wnt signaling, abundant levels of β-catenin will be dealt with by rapid and transient interactions between β-catenin and nonphosphorylated APC. In the absence of a Wnt signal, low levels of β-catenin will be tightly bound and slowly released by phosphorylated APC [21,27].

Each of the above models were challenged by a recent study in which the roles of the 20aa and 15aa repeats were addressed systematically through the functional analysis of a large number of APC variants in human cells and flies [28]. Importantly, separate roles of APC in the cytoplasmic retention and destruction of β-catenin were uncovered, involving selective APC regions. The affinities of individual β-catenin binding repeats in APC were uncovered to be of lesser importance in the destruction of β-catenin than anticipated in previous models. Instead, the β-catenin binding repeats act in concert to mediate retention of the β-catenin protein in the cytoplasm, thus preventing its activity in the nucleus. Strikingly, the second 20aa repeat which lacks affinity for β-catenin, as well as the conserved sequence B/CID, located in between the second and third 20aa repeat (Figure 1A) [28,29], perform critical roles in the APC-mediated destruction of β-catenin. How these regions control APC activity and whether this involves binding of co-factors remains to be solved.

Further experiments are needed to demonstrate if the repeat regions act simultaneously or sequentially and determine how these events are regulated by APC phosphorylation as well as by the second 20aa repeat and sequence B in the process of β-catenin destruction.

### The central domain of APC is intrinsically disordered

**Figure 2:**
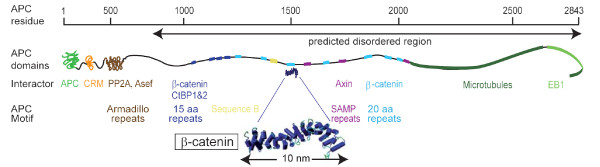
**Structural organization of the largely unfolded human APC protein**. Scaled representation of APC as a large and highly flexible protein with only a few folded segments located at its N-terminus. Folded domains, extended regions and their binding partners are indicated with a color code as in Figure 1. The extended conformation of the predicted disordered domain (F800 - V2843) is reflected by the increased protein length per amino acid residue. The relative scale indicates the maximally possible length of fully extended APC. For comparison of protein compactness, a schematic representation of the tightly folded Armadillo repeat domains of β-catenin (spanning 510 residues, comprising 10 nm only, dark blue rectangle) is depicted to scale.

Strikingly, APC lacks sequence conservation outside the small repetitive β-catenin- and axin-binding regions and sequence B. This led us to investigate the structural properties of APC in more detail using nine established algorithms to predict secondary structure and disorder. Each of the algorithms consistently indicate the presence of an exceptionally large *intrinsically disordered *region from F800 to V2843 in APC (Figure 1B) [30-35], yielding a stretch of 2000 residues with an extended, flexible conformation (Figure 2). The reliability of those algorithms is well established as illustrated using β-catenin and Axin as example proteins, for which we can compare bioinformatics results with the experimentally verified structure (Figure 1D) [36,37]. The disorder prediction algorithms for β-catenin confirm unfolded N- and C-terminal segments flanking the folded, helical core of the protein. Indeed, for these regions crystallographic studies failed to detect regular structure [36]. For the Wnt pathway tumor suppressor Axin, we experimentally confirmed the intrinsically disordered nature of the functionally important central region, thus confirming the predictions derived of various algorithms [37]. The prediction data for APC are supported by Far-UV CD spectra and NMR studies on various purified short APC fragments that include the β-catenin interacting 15aa and 20aa repeat regions in the unbound state [24]. In addition, the middle and C-terminal regions of APC, but not its N-terminus, were sensitive to proteolytic degradation, confirming a lack of globular domains in this part of the protein [38]. While short disordered sequences are common features in human proteins, such as e. g. activation loops in kinases, a continous stretch of 2000 amino acids length is rather unique in the human proteome.

Intrinsically disordered regions in proteins are rapidly gaining interest because of their intimate connection to signaling networks and their growing link to human disease [39]. Disordered regions of over 30 residues in length are 5- to 15-fold enriched in higher eukaryotic proteomes (30%) relative to eubacteria (6%) and archaea (2%), respectively [40,41], which is believed to reflect the use of disordered domains in higher order eukaryotic signaling networks. Moreover, 79% of cancer-associated proteins contain disordered regions of 30 residues or longer [42]. Among these are key tumor suppressor proteins like p53, Axin, BRCA1 and the pro-apoptotic protein ASPP2 [37,39,43].

What could be the functional advantage of such a long, extended, unstructured region for APC? Strikingly, intrinsically disordered regions are often strongly modified by e.g. phosphorylation or acetylation, which implies a relation between their dynamic appearance and regulation by posttranslational modification [44]. Indeed, such modifications can fine-tune protein-protein interactions and activity of the protein complex [24,45]. Another aspect of the extended nature of intrinsically disordered domains are simultaneous interactions with multiple binding partners with high specificity. For BRCA1, the breast cancer tumor suppressor protein, over 50 binding partners were reported, the majority of which bind to the unstructured central domain [42]. In remarkable contrast to BRCA1 and other disordered proteins, the unstructured central domain of APC mainly provides repetitive binding sites for two Wnt pathway binding partners, β-catenin and Axin.

A possible reason for this unusual property of APC might be related to the tight regulation of its activities. The main function of the central domain of APC is to downregulate cytoplasmic β-catenin levels with the assistance of Axin and its associated kinases CK1 and GSK3β. We propose that the long, flexible conformation of APC helps to sift through the cytoplasm for β-catenin substrate. The possibility to capture a β-catenin molecule is enhanced by large contact surface created by the extended peptide repeats that may allow one APC molecule to bind up to ten β-catenin molecules in the phosphorylated state (Figure 1), using four 15aa and six functional 20aa repeats. The presence of several binding sites, some of which are regulated, controls affinity for β-catenin by an avidity effect. This effect is further increased by dimerization of APC.

Disordered proteins occupy a significantly larger volume than folded proteins [46]. Assuming that the conformational ensemble of APC was random, the 2000 disordered C-terminal APC residues would occupy the volume of a particle with a diameter of 45 nm. This can be calculated based on the experimentally determined radius of gyration for disordered proteins [47], multiplied with square root of 6 [48]. Disordered proteins, however, differ from folded proteins by being able to easily adapt their shape to external influence. The structural flexibility can be illustrated by a gedankenexperiment: assuming that the backbone of one amino acid covers 3 Å in a fully extended conformation, 2000 amino acids could potentially stretch to maximally 0.6 μm when forced to do so. Its extended structure would place APC among the longest non-polymeric proteins located to the cytosol. Stretching of APC can be enforced e.g. binding to cofactors. Indeed, β-catenin was found to significantly extend the part of APC that is bound to it [21]. Extrapolation from the conformation exhibited by the disordered APC fragment in complex with β-catenin as it was found by X-Ray crystallography [21], would stretch APC to around 0.2 μm. In comparison, the folded 510 amino acid long armadillo repeat region of β-catenin, which also exhibits an extended structure, is fixed at a defined length of just about 10 nm (Figure 2). The actual length of APC is most likely between those extremes and might be modulated by its interaction partners.

### Disorder secures specificity of complex formation

In several reported cases, binding of interacting proteins induces (partial) folding or secondary structure formation of disordered regions [42,49,50]. Templates for folding include other proteins, nucleic acids, membranes, or small molecules. The conformation that is adopted by one intrinsically disordered region may differ significantly depending on the binding partner. Thus, disordered domains may provide significant binding plasticity. Therefore, disordered sequences are enriched in signaling proteins such as APC that are part of complex protein interaction networks [42].

Alternatively, the disordered regions of APC may not undergo significant conformational changes but instead remain in extended conformation to provide in abundant and transient contacts with their binding partners. As binding of a disordered protein to a partner restricts its dynamics, the interaction will involve an entropic penalty, which folded proteins do not pay [49]. As a result, disordered proteins can display highly specific interactions of relatively low affinity as compared to protein-protein interactions involving folded proteins [51]. These interactions frequently depend on electrostatic interactions tuned by posttranslational modifications (e.g. phosphorylation) that create changes in net charge of the binding regions [51,52]. Crystal structures obtained from short APC peptide fragments in complex with β-catenin [13] reveal that the phosphorylated 20aa peptide of APC does not acquire structure but remains largely extended while bound to β-catenin. The extended conformation allows merely 15 residues of APC (A1485-G1499) to bind to the entire groove on the β-catenin Arm repeat surface [21]. In contrast, the SAMP repeat segments, predicted to lack secondary structure in the unbound state, adopt a helical conformation upon binding to the Axin RGS domain [53]. Clearly, the central domain of APC uses different binding modes in its interaction with β-catenin and Axin.

How may APC exploit the properties of its unstructured domains and secure specificity of their function? Like many other scaffolding proteins, APC acts in multiple independent cellular pathways at different subcellular locations and within different protein complexes. If conformation of an unstructured domain depends on its binding partners the protein will mold to adjust to the pathway in which it functions together with signaling partners in the complex. Moreover, it can be envisioned that allosteric mechanisms propagate structure or signals to flanking domains [42,54,55], further enforcing this functionally adoptive mechanism by determining which distant binding partners are allowed to join in the protein complex. To what extend such molecular mechanisms apply for APC remains to be determined.

### Mutations in APC cause cancer

The fact that mutations in *APC *strongly predispose to colon cancer is well established [4,56]. Individuals carrying a truncating *APC *allele suffer from familial adenomatous polyposis (FAP), an autosomal dominant disorder characterized by hundreds to thousands of colorectal adenomas, some of which progress to cancer [57-59]. Moreover, mutations in APC are found in around 80% of sporadic colonic tumors [60]. The molecular events in FAP patients are phenocopied in the small intestine of mouse models in which APC is truncated after the N-terminal Armadillo repeat region, thus lacking domains involved in β-catenin downregulation [56]. The high frequency of gastro-intestinal tumor formation in these mice results from loss of the wild-type allele (LOH) and the following stabilization and accumulation of transcriptionally active nuclear β-catenin [7,8].

APC does not, however, act as a classical tumor suppressor. Careful comparison of mutations in both *Apc *alleles in tumors, levels of Wnt signaling and severity of disease in both humans and mice has led to a model in which gene dosage effects generate a defined window of enhanced Wnt signaling which leads to polyp formation in the intestine. Combinations of 'milder' *Apc *mutations, associated with weaker enhancement of Wnt signaling, rather lead to tumors in extra-intestinal tissues [61,62]. In this model, the nature of the germline mutation in *Apc *determines the type of somatic mutation mutation that occurs in the second allele. As a consequence, the resulting Wnt pathway activity is 'just right' for tumor formation [56,63].

The main focus of research in this area has been on the effects of truncating mutations in *Apc*. In these cases, large portions of the protein, including defined regulatory domains, are lost. Recent studies, using optimized technology to identify base pair alterations, indicate that in a significant number of cases, however, germline as well as sporadic single amino acid substitutions (missense mutations) in *Apc *predispose to development of colorectal adenomas [64]. Notably, a significant number of APC missense mutations were reported in tumors originating from various tissues (listed in additional file 1, Table S1, including references therein). Moreover, missense mutations in APC were linked to worse disease outcome in invasive urothelial carcinomas [65], suggesting functional relevance of point mutated APC protein in the development of extra-intestinal tumors. Most of these mutations remain functionally uncharacterized although for some missense mutant APC proteins Wnt signaling activating properties were demonstrated [66]. The molecular basis by which these mutations interfere with the function of APC remains unresolved.

### Molecular consequences of missense mutations in the disordered domain of APC

**Figure 3:**
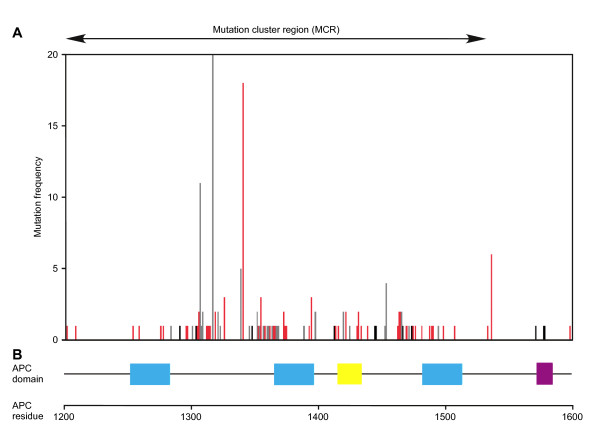
**Missense mutations cluster outside the β-catenin binding motifs in the MCR of APC**. (A) Closeup on missense mutation frequency in MCR. Color code as in Figure 1C. (B) Domain structure of the MCR, color code as in Figure 1A. We calculated mutation frequency by dividing the number of mutations in a particular codon by the total number of missense mutations reported in all referenced studies.

Long, unstructured regions are likely more apt to resist the effects of point mutations than a folded protein. In the large disordered region of APC, mutational resistance is expected to be further enhanced by multiplication of important interaction sites for partner proteins. A large number of germline and somatic missense mutations in APC however link to various forms of cancer and are identified in a scattered pattern throughout the APC protein (Figure 1C and additional file 1, Table S1). Moreover, the somatic missense mutation frequency is strongly enriched in the central Wnt regulatory MCR region that is also frequently hit by truncating mutations. Of note, the increased frequency in missense mutations could be the result of the sequencing bias of many studies in which the MCR region was selectively sequenced to identify mutations in *APC*. Remarkably, the majority of reported missense mutations in APC MCR are located outside the essential 15aa and 20aa repeat regions and their flanking N-terminal regions required for binding β-catenin (Figure 3 and additional file 1, Table S1). How can these seemingly subtle changes in an unstructured domain have such dramatic consequences? We propose several mechanisms by which missense mutations can dysregulate APC function.

(i) Altered protein interaction surface. Many of the reported missense mutations change side chain charges which might have direct implications for the formation of protein-protein interaction interfaces (additional file 1, Table S1) [66]. The single known binding partner of the MCR region is β-catenin but, surprisingly, most of the cancer-related mutations are outside the β-catenin binding repeats. Possibly, the mutated residues belong to non-redundant, so far unknown protein-protein interaction sites, although the lower conservation of these regions would render this option less likely.

(ii) Changes in secondary structure formation. Missense mutations may alter the ability of APC to adopt structure when bound to its interaction partners in the destruction complex. This would be of importance for local helix formation of APC SAMP-repeat regions upon binding Axin. Indeed, missense mutations that locate to these regions and introduce residues that reduce helix stability (Gly, Val) were reported as germline mutations in cases of adenomatous polyposis coli (Figure 3 and additional file 1, Table S1) [66-68].

(iii) Posttranslational modifications. Point mutations may interfere with posttranslational modifications of APC. For instance, mutations in APC may alter recognition sites for responsible kinases such as CK1 and GSK3β. These kinases do not directly dock onto APC but rather are positioned towards their substrate residues by binding to Axin in the complex [69]. Mutations within close proximity of target Ser residues in the 20aa repeats may interfere with phosphorylation of these motifs. Alternatively, mutations may hamper the protecting function of APC towards PP2A-mediated dephosphorylation of phospho-β-catenin and/or the delivery of β-catenin to β-TrCP [26]. Interference with this role of APC may lead to rapid dephosphorylation and stabilization of β-catenin. It is currently unknown what regions of APC are involved in these consecutive steps in β-catenin degradation and whether or not this requires extended or folded APC configuration. This information will be essential to determine whether missense mutations may interfere with this function of APC.

(iv) Dynamics of conformational equilibrium. Natively unfolded sequences may adopt a specific three dimensional conformation upon binding of a partner protein [70]. This may include a specific spatial arrangement of the repeat regions in APC. Point mutations outside those regions could prevent the required formation of a specific three-dimensional structure and, thereby, inhibit APC's usual mode of action. Hypothetically, mutations may also influence long-range intramolecular signaling. Examples of allosteric regulation of protein signaling are rapidly emerging [71]. Binding to or modification of one end of an protein elicits a signal that is communicated through the protein to trigger a response at a remote site, although it would be less obvious how such signal transmission may work in an unfolded segment.

If and how these mechanisms may play a role in the tumor suppressor activity of APC remains to be determined. It is obvious that the classical paradigms obtained for folded proteins fail to explain the phenotype of APC cancer mutations. We consider it likely that the effect is related to the dynamic nature of the disordered regions of APC. In that respect the recent discovery of the conserved sequence B as a critical functional APC unit is of interest [28,29]. Obviously, sequence B activity may involve binding partners that remain to be discovered. Alternatively, alterations in sequence B may simply disturb the dynamic interplay of the β-catenin binding repeats or modulate the competition with Axin for β-catenin binding. The importance of disordered regions is a newly emerging field, and its unusually large disordered stretch make APC a key paradigm to understand the role of unfolded regions in general.

## Conclusions and perspectives

Current mechanistic models of APC tumor suppressor function leave many questions as to how APC coordinates β-catenin degradation. Through its various domains, APC is able to interact with many different proteins. Multiple repeat regions for interaction with both β-catenin and Axin are implicated in its tumor suppressor activity. Structural information about how these proteins are positioned within the β-catenin destruction complex is lacking. Remarkably, the large central domain of APC, spanning over 2000 amino acids and carrying the repeat regions involved in β-catenin downregulation, is predicted to be entirely unstructured. This feature is rather unique in the human genome as only a few other proteins in the human proteome carry similarly sized unfolded domains. Combined structural and functional analysis of the unstructured domains of APC will be needed to reveal if structure is acquired upon binding to partner proteins. Answers as to how APC missense mutations contribute to tumorigenesis remains to be uncovered by studying how selected tumor-associated mutations interfere with essential tumor suppression mechanisms of APC.

## Competing interests

The authors declare that they have no competing interests.

## Authors' contributions

MM drafted and wrote the manuscript. SR contributed to writing of the manuscript and the generation of essential concepts. Both MM and SR supervised the project. MM, SR and DM generated the figures and contributed to the development of the concepts. DM and MM generated Table S1. ZA and DM collected and summarized essential literature and revised the manuscript critically for important and intellectual content. All authors read and approved the final manuscript.

## Supplementary Material

Additional file 1 **Table S1. Germline and somatic missense mutations in APC reported in human cancer**. List of APC missense mutations reported in human tumors. Mutated amino acid residue, affected codon, tumor type, germline or somatic nature of the mutations and corresponding references are indicated. Nucleotide numbering reflects cDNA numbering with +1 corresponding to the A of the ATG translation initiation codon in the reference sequence (Genbank NM_000038.4). The translation initiation codon is codon 1 (Genbank NP_000029). ND = Not described. *In these studies, healthy tissue was used as control. Click here for file
