# Supplementary material for: Messing up disorder: how do missense mutations in the tumor suppressor protein APC lead to cancer?
Source: Mol Cancer. 2011 Aug 22;10:101. doi: 10.1186/1476-4598-10-101 (PMC3170638; doi:10.1186/1476-4598-10-101)
Supplement: Additional file 1 — Table S1. Germline and somatic missense mutations in APC reported in human cancer. List of APC missense mutations reported in human tumors. Mutated amino acid residue, affected codon, tumor type, germline or somatic nature of the mutations and corresponding references are indicated. Nucleotide numbering reflects cDNA numbering with +1 corresponding to the A of the ATG translation initiation codon in the reference sequence (Genbank NM_000038.4). The translation initiation codon is codon 1 (Genbank NP_000029). ND = Not described. *In these studies, healthy tissue was used as control. [file 1476-4598-10-101-S1.DOC]

| **Table S1.**  **Germline and somatic missense mutations in APC reported in human cancer** | | | | | |
| --- | --- | --- | --- | --- | --- |
| **aa exchange** | **DNA**  **alteration** | **tumor type** | **somatic/- germline** | **Domain** | **Ref** |
| G53V | G158T | hamartomatous polyp | **somatic** | Homodimerization | [1]* |
| I66L | A196C | hamartomatous polyp | **somatic** |  | [1]* |
| D67H | G199C | hamartomatous polyp | **somatic** |  | [1]* |
| R99W | G295T | adenomatous polyposis coli | **germline** |  | [2] |
| R106H | G317A | adenomatous polyposis coli | **germline** |  | [3] |
| S117L | C350T | lung adenocarcinoma | **somatic** |  | [4]* |
| S127G | A379G | adenomatous polyposis coli | **germline** | Crm1 | [5] |
| S130G | A388G | malignant melanomas | **unknown** | Crm1 | [6] |
| R141S | G423T | lung adenocarcinoma | **somatic** | Crm1 | [4]* |
| K150R | A449G | colorectal adenoma | **germline** | Crm1 | [3] |
| S171I | G512T | adenomatous polyposis coli | **germline** | Crm1 | [7] |
| A199T | G595A | adenomatous polyposis coli | **germline** | Crm1 | [3] |
| Q208R | A623G | adenomatous polyposis coli | **germline** | Crm1 | [8] |
| R216Q | G647A | colorectal adenoma | **germline** | Crm1 | [3] |
| E241K | G721A | adenomatous polyposis coli | **germline** | Crm1 | [9] |
| E251K | G836A | breast cancer (HR+) | **somatic** |  | [10]* |
| R267L | G800T | adenomatous polyposis coli | **somatic** |  | [11] |
| R380P | G1139C | hamartomatous polyp | **somatic** |  | [1]* |
| R414C | C1240T | adenomatous polyposis coli | **germline** |  | [11] |
| H464Q | T1392A | hamartomatous polyp | **somatic** | Armadillo | [1]* |
| A465S | G1393T | hamartomatous polyp | **somatic** | Armadillo | [1]* |
| S490L | C1469T | adenomatous polyposis coli | **somatic** | Armadillo | [11] |
| A501V | C1502T | adenomatous polyposis coli | **germline** | Armadillo | [12] |
| R506Q | G1517A | adenomatous polyposis coli | **somatic** | Armadillo | [11] |
| K516N | G1548C | adenomatous polyposis coli | **germline** | Armadillo | [13] |
| S537C | A1609T | adenomatous polyposis coli | **germline** | Armadillo | [3] |
| E538V | A1613T | colorectal adenoma | **germline** | Armadillo | [3] |
| L639S | T1916C | adenomatous polyposis coli | **germline** | Armadillo | [3] |
| S643P | T1927C | colorectal adenoma | **germline** | Armadillo | [3] |
| R653K | G1958A | colorectal adenoma | **germline** | Armadillo | [3] |
| L662V | C1984G | lung adenocarcinoma | **somatic** | Armadillo | [4]* |
| A698V | C2093T | adenomatous polyposis coli | **somatic** | Armadillo | [11] |
| S722G | A2164G | adenomatous polyposis coli | **germline** | Armadillo | [14] |
| S784T | T2350A | adenomatous polyposis coli | **germline** |  | [15] |
| N813S | A2438G | malignant melanomas | **unknown** |  | [6] |
| G817C | G2449T | gastric cancer | **somatic** |  | [16] |
| L852M | T2554A | sporadic colorectal carcinomas | **somatic** |  | [17]* |
| P865S | C2593T | colorectal tumour | **unknown** |  | [18] |
| P870S | C2608T | colorectal adenoma | **germline** |  | [3] |
| I880T | T2639C | gastric cancer | **somatic** |  | [16] |
| I880T | T2639C | colorectal tumour | **somatic** |  | [19]* |
| V890I | G2668A | colorectal tumour | **somatic** |  | [19]* |
| S906Y | C2717A | early colorectal tumour | **somatic** |  | [20]* |
| E911G | A2732G | colorectal tumour | **somatic** |  | [21]* |
| N942D | A2824G | gastric cancer | **somatic** |  | [16] |
| C947S | G2840C | colorectal adenoma | **germline** |  | [3] |
| M949I | G2847T | colorectal adenoma | **germline** |  | [3] |
| S966R | A2896C | sporadic colorectal carcinoma | **somatic** |  | [17]* |
| P981R | C2942G | colorectal adenoma | **germline** |  | [3] |
| N1026S | A3077G | adenomatous polyposis coli, attenuated | **germline** | 15 aa repeat 1 | [22] |
| Y1027C | A3080G | desmoid tumour | **somatic** | 15 aa repeat 1 | [23]* |
| D1057G | A3170G | adenomatous polyposis coli | **germline** |  | [24] |
| S1081I | G3242T | breast carcinoma | **somatic** |  | [25] |
| Y1102H | T3304C | sporadic colorectal carcinoma | **somatic** |  | [17]* |
| N1118D | A3352G | adenomatous polyposis coli | **germline** |  | [9] |
| G1120E | G3359A | gastric cancer | **somatic** |  | [26] |
| V1125A | T3374C | colorectal adenoma | **germline** |  | [3] |
| L1129S | T3386C | adenomatous polyposis coli | **germline** |  | [9] |
| L1129S | T3386C | colorectal cancer | **somatic** |  | [27]* |
| T1160K | C3479A | colorectal adenoma | **germline** | 15 aa repeat 3 | [3] |
| R1171H | G3512A | gastric cancer | **somatic** |  | [16] |
| P1176L | C3527T | adenomatous polyposis coli | **germline** | 15 aa repeat 4 | [28] |
| A1184P | G3550C | adenomatous polyposis coli | **germline** | 15 aa repeat 4 | [29] |
| F1197S | T3590C | gastric cancer | **somatic** |  | [16] |
| S1202C | C3605G | intestinal-type gastric cancer | **somatic** |  | [30]* |
| E1209V | A3626T | lung adenocarcinoma | **somatic** |  | [4]* |
| I1254F | A3760T | colorectal tumour | **somatic** |  | [31]* |
| I1259T | T3776C | gastric cancer | **somatic** | 20 aa repeat 1 | [16] |
| S1276L | C3827T | breast cancer (her2 +) | **somatic** | 20 aa repeat 1 | [10]* |
| S1278P | T3832C | urothelial carcinoma | **somatic** | 20 aa repeat 1 | [32]* |
| E1284K | G3850A | lung cancer | **somatic** | 20 aa repeat 1 | [33] |
| Q1291E | C3871G | adenomatous polyposis coli | **germline** |  | [34] |
| A1296V | C3887T | cerebellar medulloblastoma | **somatic** |  | [35]* |
| D1297A | A3890C | colorectal carcinoma | **somatic** |  | [36] |
| T1301S | C3902G | early gastric tumour | **unknown** |  | [37] |
| I1304V | A3910G | juveniIe polyposis coli | **unknown** |  | [38] |
| A1305G | C3914G | colorectal cancer | **somatic** |  | [39]* |
| E1306K | G3916A | hepatoblastoma | **somatic** |  | [40]* |
| E1306K | G3916A | ampullary carcinoma | **somatic** |  | [41] |
| I1307K | T3920A | colorectal cancer | **germline** |  | [42] |
| I1307K | T3920A | colorectal cancer | **somatic** |  | [39]* |
| I1307K | ND | early-onset colorectal carcinoma | **unknown** |  | [43] |
| K1308E | A3922G | serrated adenoma | **unknown** |  | [44] |
| E1309G | A3926G | juveniIe polyposis coli | **unknown** |  | [38] |
| E1309K | G3925A | intestinal-type adenocarcinoma | **unknown** |  | [45] |
| G1312E | G3935A | gastric cancer | **somatic** |  | [16] |
| T1313A | A3937G | colorectal tumor | **somatic** |  | [21]* |
| R1314K | G3941A | ampullary carcinoma | **somatic** |  | [41] |
| S1315L | C3944T | colorectal cancer | **somatic** |  | [46]* |
| E1317Q | G3949C | colorectal cancer | **germline** |  | [47] |
| E1317Q | G3949C | colorectal tumour | **somatic** |  | [48] |
| E1317Q | G3949C | sporadic colorectal carcinoma | **somatic** |  | [17]* |
| E1317Q | G3949C | lung cancer | **somatic** |  | [33] |
| P1319T | C3955A | genesis of hepatoblastoma | **somatic** |  | [40]* |
| P1319L | C3956T | neuroectodermal tumour | **somatic** |  | [49]* |
| S1321R | C3963A | pancreatic carcinoma | **unknown** |  | [50] |
| S1321N | G3962A | genesis of hepatoblastoma | **somatic** |  | [40]* |
| V1323I | ND | colorectal cancer | **unknown** |  | [51] |
| V1326A | T3977C | gastric cancer | **somatic** |  | [16] |
| V1326M | G3976A | genesis of hepatoblastoma | **somatic** |  | [40]* |
| V1326M | G3976A | genesis of hepatoblastoma | **somatic** |  | [40]* |
| G1339A | G4016C | ameloblastoma , odontogenic carcinoma | **unknown** |  | [52] |
| G1339A | G4016C | ameloblastoma and odontogenic carcinoma | **unknown** |  | [52] |
| G1339A | G4016C | ameloblastoma and odontogenic carcinoma | **unknown** |  | [52] |
| G1339A | G4016C | ameloblastoma and odontogenic carcinoma | **unknown** |  | [52] |
| G1339A | G4016C | ameloblastoma and odontogenic carcinoma | **unknown** |  | [52] |
| S1341R | T4023G | desmoid-type fibromatosis | **somatic** |  | [53] |
| S1341R | T4023G | desmoid-type fibromatosis | **somatic** |  | [53] |
| S1341R | T4023G | desmoid-type fibromatosis | **somatic** |  | [53] |
| S1341R | T4023G | desmoid-type fibromatosis | **somatic** |  | [53] |
| S1341R | T4023G | desmoid-type fibromatosis | **somatic** |  | [53] |
| S1341R | T4023G | desmoid-type fibromatosis | **somatic** |  | [53] |
| S1341R | T4023G | desmoid-type fibromatosis | **somatic** |  | [53] |
| S1341R | T4023G | desmoid-type fibromatosis | **somatic** |  | [53] |
| S1341R | T4023G | desmoid-type fibromatosis | **somatic** |  | [53] |
| S1341R | T4023G | aggressive fibromatosis | **somatic** |  | [54] |
| S1341R | T4023G | aggressive fibromatosis | **somatic** |  | [54] |
| S1341R | T4023G | aggressive fibromatosis | **somatic** |  | [54] |
| S1341R | T4023G | aggressive fibromatosis | **somatic** |  | [54] |
| S1341R | T4023G | aggressive fibromatosis | **somatic** |  | [54] |
| S1341R | T4023G | aggressive fibromatosis | **somatic** |  | [54] |
| S1341R | T4023G | aggressive fibromatosis | **somatic** |  | [54] |
| S1341R | T4023G | aggressive fibromatosis | **somatic** |  | [54] |
| S1341R | T4023G | aggressive fibromatosis | **somatic** |  | [54] |
| S1346P | T4036C | thyroid tumour | **unknown** |  | [55] |
| R1348W | A4042T | adenomatous polyposis coli | **germline** |  | [28] |
| V1352A | T4055C | colorectal adenoma | **germline** |  | [3] |
| V1352I | G4054A | urothelial carcinoma | **somatic** |  | [32]* |
| E1353V | A4058T | non-polypoid colorectal tumour | **somatic** |  | [56]* |
| F1354L | T4062A | colorectal tumour | **unknown** |  | [57] |
| S1355P | T4063C | colorectal tumour | **somatic** |  | [58]* |
| S1355P | T4063C | desmoid-type fibromatosis | **somatic** |  | [53] |
| S1355P | T4063C | aggressive fibromatosis | **somatic** |  | [54] |
| G1357R | G4069A | colorectal tumour | **unknown** |  | [57] |
| A1358E | C4073A | duodenal adenoma | **somatic** |  | [59] |
| S1360F | ND | colorectal cancer | **unknown** |  | [51] |
| P1361L | C4082T | alveolar soft part sarcoma | **unknown** |  | [60] |
| S1362F | C4085T | early colorectal cancer | **somatic** |  | [61]* |
| S1364R | T4092G | colorectal tumour | **unknown** |  | [62] |
| G1365D | G4094A | urothelial carcinoma | **somatic** |  | [32]* |
| A1366V | C4097T | colorectal carcinoma | **somatic** |  | [36] |
| Q1367H | G4101C | colorectal tumour | **unknown** |  | [57] |
| T1368I | ND | Medulloblastoma | **unknown** | 20 aa repeat 2 | [63] |
| P1369H | ND | Medulloblastoma | **unknown** | 20 aa repeat 2 | [63] |
| P1373T | C4117A | non-polypoid colorectal tumour | **somatic** | 20 aa repeat 2 | [56]* |
| P1373S | C4117T | urothelial carcinoma | **somatic** | 20 aa repeat 2 | [32]* |
| E1374K | G4120A | ampullary carcinoma | **somatic** | 20 aa repeat 2 | [41] |
| H1375Y | C4123T | ampullary carcinoma | **somatic** | 20 aa repeat 2 | [41] |
| S1389F | C4166T | colorectal tumour | **unknown** | 20 aa repeat 2 | [57] |
| L1393P | T4178C | non-polypoid colorectal tumour | **somatic** | 20 aa repeat 2 | [56]* |
| S1395C | A4183T | hepatoblastoma | **somatic** | 20 aa repeat 2 | [40]* |
| S1395C | A4183T | hepatoblastoma | **somatic** | 20 aa repeat 2 | [40]* |
| S1395C | A4183T | hepatoblastoma | **somatic** | 20 aa repeat 2 | [40]* |
| S1398N | G4193A | esophageal and esophagogastric adenocarcinoma | **somatic** |  | [64]* |
| S1398T | G4193C | colorectal tumour | **unknown** |  | [57] |
| M1413V | A4237G | colorectal adenoma | **germline** |  | [3] |
| V1414I | G4240A | endocrine tumour | **somatic** |  | [65]* |
| G1416D | G4247A | endocrine tumour | **somatic** | Sequence B | [65]* |
| P1420L | C4259T | intestinal-type adenocarcinoma | **unknown** | Sequence B | [45] |
| P1420L | C4259T | intestinal-type adenocarcinoma | **unknown** | Sequence B | [45] |
| D1422H | G4264C | desmoid tumour | **somatic** | Sequence B | [23] |
| D1422N | G4264A | endocrine tumours | **somatic** | Sequence B | [65]* |
| D1425A | A4274C | colorectal tumour | **unknown** | Sequence B | [62] |
| M1431R | T4292G | colorectal cancer | **somatic** | Sequence B | [39]* |
| P1432H | CA4295AC | desmoid-type fibromatosis | **somatic** | Sequence B | [53] |
| P1432H | CA4295AC | aggressive fibromatosis | **somatic** | Sequence B | [54] |
| S1434I | G4301T | colorectal tumour | **somatic** | Sequence B | [66]* |
| P1439L | C4316T | endocrine tumour | **somatic** |  | [65]* |
| T1445A | A4333G | colorectal adenoma | **germline** |  | [3] |
| A1446T | G4336A | colorectal adenoma | **germline** |  | [3] |
| P1453S | C4357T | intestinal-type adenocarcinoma | **unknown** |  | [45] |
| K1454E | A4360G | colorectal adenoma | **germline** |  | [3] |
| K1454E | A4360G | urothelial carcinoma | **somatic** |  | [32]* |
| K1454E | A4360G | urothelial carcinoma | **somatic** |  | [32]* |
| K1454E | A4360G | urothelial carcinoma | **somatic** |  | [32]* |
| R1463S | A4389T | intestinal-type gastric cancer | **somatic** |  | [30]* |
| E1464K | G4390A | endocrine tumour | **somatic** |  | [65]* |
| E1464D | G4392C | urothelial carcinoma | **somatic** |  | [32]* |
| S1465R | T4395G | colorectal cancer | **somatic** |  | [39]* |
| G1466E | G4397A | intestinal-type adenocarcinoma | **unknown** |  | [45] |
| G1466R | G4396A | urothelial carcinoma | **somatic** |  | [32]* |
| P1467S | C4399T | colorectal adenoma | **germline** |  | [3] |
| A1470T | G4408A | early gastric carcinoma | **somatic** |  | [67] |
| V1472I | G4414A | cerebellar medulloblastoma | **unknown** |  | [35]* |
| A1474T | G4420A | colorectal adenoma | **germline** |  | [3] |
| A1475V | C4424T | urothelial carcinoma | **somatic** |  | [32]* |
| Q1477H | G4431T | malignant melanoma | **somatic** |  | [68] |
| L1482F | C4444T | endocrine tumour | **somatic** |  | [65]* |
| L1488F | A4464T | colorectal cancer | **somatic** | 20 aa repeat 3 | [39]* |
| H1490Y | C4468T | urothelial carcinoma | **somatic** | 20 aa repeat 3 | [32]* |
| F1491L | T4471C | colorectal cancer | **somatic** | 20 aa repeat 3 | [46]* |
| S1495G | A4483G | cerebellar medulloblastoma | **unknown** | 20 aa repeat 3 | [35]* |
| G1499R | G4495A | ampullary carcinoma | **somatic** | 20 aa repeat 3 | [41] |
| A1508V | C4523T | colorectal tumor | **somatic** | 20 aa repeat 3 | [19]* |
| G1534R | G4600A | hepatoblastoma | **somatic** |  | [40]* |
| T1537K | C4610A | desmoid-type fibromatosis | **somatic** |  | [53] |
| T1537K | C4610A | desmoid-type fibromatosis | **somatic** |  | [53] |
| T1537K | C4610A | desmoid-type fibromatosis | **somatic** |  | [53] |
| T1537K | C4610A | aggressive fibromatosis | **somatic** |  | [54] |
| T1537K | C4610A | aggressive fibromatosis | **somatic** |  | [54] |
| T1537K | C4610A | aggressive fibromatosis | **somatic** |  | [54] |
| I1572T | T4715C | colorectal adenoma | **germline** |  | [3] |
| C1578G | T4732G | colorectal adenoma | **germline** | SAMP repeat 1 | [3] |
| I1579V | A4735G | colorectal adenoma | **germline** | SAMP repeat 1 | [3] |
| S1599L | C4796T | colorectal tumour | **somatic** |  | [69]* |
| P1609L | C4826T | Glioblastoma | **somatic** |  | [70]* |
| R1640W | C4918T | adenomatous polyposis coli | **germline** | 20 aa repeat 4 | [14] |
| R1676G | A5026G | adenomatous polyposis coli | **germline** |  | [3] |
| D1714N | G5140A | adenomatous polyposis coli | **germline** |  | [9] |
| D1822V | A5465T | adenomatous polyposis coli | **germline** |  | [24] |
| P1843L | C5528T | adenomatous polyposis coli | **germline** | 20 aa repeat 5 | [71] |
| G1921S | G5761A | colorectal adenoma | **germline** |  | [3] |
| P1934L | C5801T | colorectal adenoma | **germline** |  | [3] |
| S1971C | C5912G | adenomatous polyposis coli | **germline** | 20 aa repeat 6 | [72] |
| L2038V | C6112G | adenomatous polyposis coli | **germline** | SAMP repeat 3 | [73] |
| R2066G | A6197G | colorectal adenoma | **germline** | Microtubules | [3] |
| H2149P | A6446C | adenomatous polyposis coli | **germline** | Microtubules | [3] |
| P2158R | C6473G | colorectal adenoma | **germline** | Microtubules | [3] |
| H2232D | C6694G | colorectal adenoma | **germline** | Microtubules | [3] |
| A2274V | C6821T | adenomatous polyposis coli | **germline** | Microtubules | [3] |
| I2329V | A6985G | colorectal adenoma | **germline** | Microtubules | [3] |
| P2467T | C7399A | adenomatous polyposis coli | **germline** | Microtubules | [3] |
| G2502S | G7504A | adenomatous polyposis coli | **germline** | Microtubules | [74] |
| R2505Q | G7514A | adenomatous polyposis coli | **germline** | Microtubules | [9] |
| P2540S | C7618T | malignant melanoma | **unknown** | Microtubules | [6] |
| I2541V | A7621G | colorectal adenoma | **germline** | Microtubules | [3] |
| R2566K | G7697A | adenomatous polyposis coli | **germline** | Microtubules | [75] |
| S2570L | C7709T | renal carcinoma | **somatic** | Microtubules | [76]* |
| I2573V | A7717G | colorectal adenoma | **germline** | Microtubules | [3] |
| N2593S | A7778G | colorectal adenoma | **germline** | Microtubules | [3] |
| S2621C | C7862G | adenomatous polyposis coli | **germline** | Microtubules | [15] |
| S2631F | C7892T | lung adenocarcinoma | **somatic** | Microtubules | [4]* |
| A2690T | G8068A | adenomatous polyposis coli | **germline** | Microtubules | [9] |
| I2738T | T8213C | adenomatous polyposis coli | **germline** | Microtubules | [24] |
| I2756V | A8266G | colorectal adenoma | **germline** | Microtubules | [3] |
| A2795T | G8383A | colorectal adenoma | **germline** | EB1 | [3] |
| S2822N | G8465A | lung adenocarcinoma | **somatic** | EB1 | [10]* |
| L2839F | C8515T | adenomatous polyposis coli | **germline** | Microtubules | [15] |

Table S1. APC missense mutations described in human tumors. Nucleotide numbering reflects cDNA numbering with +1 corresponding to the A of the ATG translation initiation codon in the reference sequence (Genbank NM_000038.4). The translation initiation codon is codon 1 (Genbank NP_000029).

ND = Not described

*In these studies, healthy tissue was used as control.

**References Additional file 1, Table S1**

1. Kim JC, Roh SA, Kim HC, Yu CS, Lee DH, et al. (1999) Somatic mutations of the first 14 exons of APC in hamartomatous polyps of the colon. Hum Mutat 14: 351-352.

2. Dobbie Z, Spycher M, Hurliman R, Ammann R, Ammann T, et al. (1994) Mutational analysis of the first 14 exons of the adenomatous polyposis coli (APC) gene. Eur J Cancer 30A: 1709-1713.

3. Azzopardi D, Dallosso AR, Eliason K, Hendrickson BC, Jones N, et al. (2008) Multiple rare nonsynonymous variants in the adenomatous polyposis coli gene predispose to colorectal adenomas. Cancer Res 68: 358-363.

4. Ding L, Getz G, Wheeler DA, Mardis ER, McLellan MD, et al. (2008) Somatic mutations affect key pathways in lung adenocarcinoma. Nature 455: 1069-1075.

5. Al-Sukhni W, Aronson M, Gallinger S (2008) Hereditary colorectal cancer syndromes: familial adenomatous polyposis and lynch syndrome. Surg Clin North Am 88: 819-844, vii.

6. Worm J, Christensen C, Gronbaek K, Tulchinsky E, Guldberg P (2004) Genetic and epigenetic alterations of the APC gene in malignant melanoma. Oncogene 23: 5215-5226.

7. van der Luijt RB, Khan PM, Vasen HF, Tops CM, van Leeuwen-Cornelisse IS, et al. (1997) Molecular analysis of the APC gene in 105 Dutch kindreds with familial adenomatous polyposis: 67 germline mutations identified by DGGE, PTT, and southern analysis. Hum Mutat 9: 7-16.

8. Ficari F, Cama A, Valanzano R, Curia MC, Palmirotta R, et al. (2000) APC gene mutations and colorectal adenomatosis in familial adenomatous polyposis. Br J Cancer 82: 348-353.

9. Zhou XL, Eriksson U, Werelius B, Kressner U, Sun XF, et al. (2004) Definition of candidate low risk APC alleles in a Swedish population. Int J Cancer 110: 550-557.

10. Kan Z, Jaiswal BS, Stinson J, Janakiraman V, Bhatt D, et al. (2010) Diverse somatic mutation patterns and pathway alterations in human cancers. Nature 466: 869-873.

11. Nishisho I, Nakamura Y, Miyoshi Y, Miki Y, Ando H, et al. (1991) Mutations of chromosome 5q21 genes in FAP and colorectal cancer patients. Science 253: 665-669.

12. Wu G, Wu W, Hegde M, Fawkner M, Chong B, et al. (2001) Detection of sequence variations in the adenomatous polyposis coli (APC) gene using denaturing high-performance liquid chromatography. Genet Test 5: 281-290.

13. Cowie S, Drmanac S, Swanson D, Delgrosso K, Huang S, et al. (2004) Identification of APC gene mutations in colorectal cancer using universal microarray-based combinatorial sequencing-by-hybridization. Hum Mutat 24: 261-271.

14. Stella A, Montera M, Resta N, Marchese C, Susca F, et al. (1994) Four novel mutations of the APC (adenomatous polyposis coli) gene in FAP patients. Hum Mol Genet 3: 1687-1688.

15. Miyoshi Y, Ando H, Nagase H, Nishisho I, Horii A, et al. (1992) Germ-line mutations of the APC gene in 53 familial adenomatous polyposis patients. Proc Natl Acad Sci U S A 89: 4452-4456.

16. Nakatsuru S, Yanagisawa A, Ichii S, Tahara E, Kato Y, et al. (1992) Somatic mutation of the APC gene in gastric cancer: frequent mutations in very well differentiated adenocarcinoma and signet-ring cell carcinoma. Hum Mol Genet 1: 559-563.

17. Lovig T, Meling GI, Diep CB, Thorstensen L, Norheim Andersen S, et al. (2002) APC and CTNNB1 mutations in a large series of sporadic colorectal carcinomas stratified by the microsatellite instability status. Scand J Gastroenterol 37: 1184-1193.

18. Domingo E, Espin E, Armengol M, Oliveira C, Pinto M, et al. (2004) Activated BRAF targets proximal colon tumors with mismatch repair deficiency and MLH1 inactivation. Genes Chromosomes Cancer 39: 138-142.

19. Miyaki M, Nishio J, Konishi M, Kikuchi-Yanoshita R, Tanaka K, et al. (1997) Drastic genetic instability of tumors and normal tissues in Turcot syndrome. Oncogene 15: 2877-2881.

20. Powell SM, Zilz N, Beazer-Barclay Y, Bryan TM, Hamilton SR, et al. (1992) APC mutations occur early during colorectal tumorigenesis. Nature 359: 235-237.

21. Miyoshi Y, Nagase H, Ando H, Horii A, Ichii S, et al. (1992) Somatic mutations of the APC gene in colorectal tumors: mutation cluster region in the APC gene. Hum Mol Genet 1: 229-233.

22. Menendez M, Gonzalez S, Obrador-Hevia A, Dominguez A, Pujol MJ, et al. (2008) Functional characterization of the novel APC N1026S variant associated with attenuated familial adenomatous polyposis. Gastroenterology 134: 56-64.

23. Miyaki M, Konishi M, Kikuchi-Yanoshita R, Enomoto M, Tanaka K, et al. (1993) Coexistence of somatic and germ-line mutations of APC gene in desmoid tumors from patients with familial adenomatous polyposis. Cancer Res 53: 5079-5082.

24. Wallis YL, Morton DG, McKeown CM, Macdonald F (1999) Molecular analysis of the APC gene in 205 families: extended genotype-phenotype correlations in FAP and evidence for the role of APC amino acid changes in colorectal cancer predisposition. J Med Genet 36: 14-20.

25. Kashiwaba M, Tamura G, Ishida M (1994) Aberrations of the APC gene in primary breast carcinoma. J Cancer Res Clin Oncol 120: 727-731.

26. Horii A, Nakatsuru S, Miyoshi Y, Ichii S, Nagase H, et al. (1992) The APC gene, responsible for familial adenomatous polyposis, is mutated in human gastric cancer. Cancer Res 52: 3231-3233.

27. Rapozo DC, Grinmann AB, Carvalho AT, de Souza HS, Soares-Lima SC, et al. (2009) Analysis of mutations in TP53, APC, K-ras, and DCC genes in the non-dysplastic mucosa of patients with inflammatory bowel disease. Int J Colorectal Dis 24: 1141-1148.

28. Nagase H, Miyoshi Y, Horii A, Aoki T, Petersen GM, et al. (1992) Screening for germ-line mutations in familial adenomatous polyposis patients: 61 new patients and a summary of 150 unrelated patients. Hum Mutat 1: 467-473.

29. Lamlum H, Ilyas M, Rowan A, Clark S, Johnson V, et al. (1999) The type of somatic mutation at APC in familial adenomatous polyposis is determined by the site of the germline mutation: a new facet to Knudson's 'two-hit' hypothesis. Nat Med 5: 1071-1075.

30. Ebert MP, Fei G, Kahmann S, Muller O, Yu J, et al. (2002) Increased beta-catenin mRNA levels and mutational alterations of the APC and beta-catenin gene are present in intestinal-type gastric cancer. Carcinogenesis 23: 87-91.

31. Sjoblom T, Jones S, Wood LD, Parsons DW, Lin J, et al. (2006) The consensus coding sequences of human breast and colorectal cancers. Science 314: 268-274.

32. Kastritis E, Murray S, Kyriakou F, Horti M, Tamvakis N, et al. (2009) Somatic mutations of adenomatous polyposis coli gene and nuclear b-catenin accumulation have prognostic significance in invasive urothelial carcinomas: evidence for Wnt pathway implication. Int J Cancer 124: 103-108.

33. Ohgaki H, Kros JM, Okamoto Y, Gaspert A, Huang H, et al. (2004) APC mutations are infrequent but present in human lung cancer. Cancer Lett 207: 197-203.

34. Gavert N, Yaron Y, Naiman T, Bercovich D, Rozen P, et al. (2002) Molecular analysis of the APC gene in 71 Israeli families: 17 novel mutations. Hum Mutat 19: 664.

35. Huang H, Mahler-Araujo BM, Sankila A, Chimelli L, Yonekawa Y, et al. (2000) APC mutations in sporadic medulloblastomas. Am J Pathol 156: 433-437.

36. Prall F, Weirich V, Ostwald C (2007) Phenotypes of invasion in sporadic colorectal carcinomas related to aberrations of the adenomatous polyposis coli (APC ) gene. Histopathology 50: 318-330.

37. Tamura G, Maesawa C, Suzuki Y, Tamada H, Satoh M, et al. (1994) Mutations of the APC gene occur during early stages of gastric adenoma development. Cancer Res 54: 1149-1151.

38. Kim JC, Roh SA, Yu CS, Lee HI, Gong G (1997) Familial juvenile polyposis coli with APC gene mutation. Am J Gastroenterol 92: 1913-1915.

39. Kamory E, Olasz J, Csuka O (2008) Somatic APC inactivation mechanisms in sporadic colorectal cancer cases in Hungary. Pathol Oncol Res 14: 51-56.

40. Oda H, Imai Y, Nakatsuru Y, Hata J, Ishikawa T (1996) Somatic mutations of the APC gene in sporadic hepatoblastomas. Cancer Res 56: 3320-3323.

41. Imai Y, Oda H, Tsurutani N, Nakatsuru Y, Inoue T, et al. (1997) Frequent somatic mutations of the APC and p53 genes in sporadic ampullary carcinomas. Jpn J Cancer Res 88: 846-854.

42. Laken SJ, Petersen GM, Gruber SB, Oddoux C, Ostrer H, et al. (1997) Familial colorectal cancer in Ashkenazim due to a hypermutable tract in APC. Nat Genet 17: 79-83.

43. Yantiss RK, Goodarzi M, Zhou XK, Rennert H, Pirog EC, et al. (2009) Clinical, pathologic, and molecular features of early-onset colorectal carcinoma. Am J Surg Pathol 33: 572-582.

44. Dehari R (2001) Infrequent APC mutations in serrated adenoma. Tohoku J Exp Med 193: 181-186.

45. Frattini M, Perrone F, Suardi S, Balestra D, Caramuta S, et al. (2006) Phenotype-genotype correlation: challenge of intestinal-type adenocarcinoma of the nasal cavity and paranasal sinuses. Head Neck 28: 909-915.

46. Bougatef K, Ouerhani S, Moussa A, Kourda N, Coulet F, et al. (2008) Prevalence of mutations in APC, CTNNB1, and BRAF in Tunisian patients with sporadic colorectal cancer. Cancer Genet Cytogenet 187: 12-18.

47. Frayling IM, Beck NE, Ilyas M, Dove-Edwin I, Goodman P, et al. (1998) The APC variants I1307K and E1317Q are associated with colorectal tumors, but not always with a family history. Proc Natl Acad Sci U S A 95: 10722-10727.

48. Rowan AJ, Lamlum H, Ilyas M, Wheeler J, Straub J, et al. (2000) APC mutations in sporadic colorectal tumors: A mutational "hotspot" and interdependence of the "two hits". Proc Natl Acad Sci U S A 97: 3352-3357.

49. Koch A, Waha A, Tonn JC, Sorensen N, Berthold F, et al. (2001) Somatic mutations of WNT/wingless signaling pathway components in primitive neuroectodermal tumors. Int J Cancer 93: 445-449.

50. Yashima K, Nakamori S, Murakami Y, Yamaguchi A, Hayashi K, et al. (1994) Mutations of the adenomatous polyposis coli gene in the mutation cluster region: comparison of human pancreatic and colorectal cancers. Int J Cancer 59: 43-47.

51. Sanchez-de-Abajo A, de la Hoya M, van Puijenbroek M, Tosar A, Lopez-Asenjo JA, et al. (2007) Molecular analysis of colorectal cancer tumors from patients with mismatch repair proficient hereditary nonpolyposis colorectal cancer suggests novel carcinogenic pathways. Clin Cancer Res 13: 5729-5735.

52. Siriwardena BS, Kudo Y, Ogawa I, Tilakaratne WM, Takata T (2009) Aberrant beta-catenin expression and adenomatous polyposis coli gene mutation in ameloblastoma and odontogenic carcinoma. Oral Oncol 45: 103-108.

53. Jilong Y, Jian W, Xiaoyan Z, Xiaoqiu L, Xiongzeng Z (2007) Analysis of APC/beta-catenin genes mutations and Wnt signalling pathway in desmoid-type fibromatosis. Pathology 39: 319-325.

54. Yang JL, Wang J, Zhou XY, Zhu XZ (2008) [Abnormalities of chromosome 8, APC and beta-catenin genes in aggressive fibromatosis]. Zhonghua Zhong Liu Za Zhi 30: 116-120.

55. Zeki K, Spambalg D, Sharifi N, Gonsky R, Fagin JA (1994) Mutations of the adenomatous polyposis coli gene in sporadic thyroid neoplasms. J Clin Endocrinol Metab 79: 1317-1321.

56. Umetani N, Sasaki S, Masaki T, Watanabe T, Matsuda K, et al. (2000) Involvement of APC and K-ras mutation in non-polypoid colorectal tumorigenesis. Br J Cancer 82: 9-15.

57. Yuan P, Sun MH, Zhang JS, Zhu XZ, Shi DR (2001) APC and K-ras gene mutation in aberrant crypt foci of human colon. World J Gastroenterol 7: 352-356.

58. Suzui M, Yoshimi N, Hara A, Morishita Y, Tanaka T, et al. (1998) Genetic alterations in a patient with Turcot's syndrome. Pathol Int 48: 126-133.

59. Norheim Andersen S, Lovig T, Fausa O, Rognum TO (1999) Germline and somatic mutations in exon 15 of the APC gene and K-ras mutations in duodenal adenomas in patients with familial adenomatous polyposis. Scand J Gastroenterol 34: 611-617.

60. Saito T, Oda Y, Kawaguchi K, Takahira T, Yamamoto H, et al. (2003) Possible association between tumor-suppressor gene mutations and hMSH2/hMLH1 inactivation in alveolar soft part sarcoma. Hum Pathol 34: 841-849.

61. Nielsen M, de Miranda NF, van Puijenbroek M, Jordanova ES, Middeldorp A, et al. (2009) Colorectal carcinomas in MUTYH-associated polyposis display histopathological similarities to microsatellite unstable carcinomas. BMC Cancer 9: 184.

62. Ogasawara S, Maesawa C, Tamura G, Satodate R (1994) Lack of mutations of the adenomatous polyposis coli gene in oesophageal and gastric carcinomas. Virchows Arch 424: 607-611.

63. Thompson MC, Fuller C, Hogg TL, Dalton J, Finkelstein D, et al. (2006) Genomics identifies medulloblastoma subgroups that are enriched for specific genetic alterations. J Clin Oncol 24: 1924-1931.

64. Choi YW, Heath EI, Heitmiller R, Forastiere AA, Wu TT (2000) Mutations in beta-catenin and APC genes are uncommon in esophageal and esophagogastric junction adenocarcinomas. Mod Pathol 13: 1055-1059.

65. Pizzi S, Azzoni C, Tamburini E, Bottarelli L, Campanini N, et al. (2008) Adenomatous polyposis coli alteration in digestive endocrine tumours: correlation with nuclear translocation of beta-catenin and chromosomal instability. Endocr Relat Cancer 15: 1013-1024.

66. Homfray TF, Cottrell SE, Ilyas M, Rowan A, Talbot IC, et al. (1998) Defects in mismatch repair occur after APC mutations in the pathogenesis of sporadic colorectal tumours. Hum Mutat 11: 114-120.

67. Nakatsuru S, Yanagisawa A, Furukawa Y, Ichii S, Kato Y, et al. (1993) Somatic mutations of the APC gene in precancerous lesion of the stomach. Hum Mol Genet 2: 1463-1465.

68. Reifenberger J, Knobbe CB, Wolter M, Blaschke B, Schulte KW, et al. (2002) Molecular genetic analysis of malignant melanomas for aberrations of the WNT signaling pathway genes CTNNB1, APC, ICAT and BTRC. Int J Cancer 100: 549-556.

69. Blaker H, Scholten M, Sutter C, Otto HF, Penzel R (2003) Somatic mutations in familial adenomatous polyps. Nuclear translocation of beta-catenin requires more than biallelic APC inactivation. Am J Clin Pathol 120: 418-423.

70. (2008) Comprehensive genomic characterization defines human glioblastoma genes and core pathways. Nature 455: 1061-1068.

71. Hadjisavvas A, Papasavva T, Loizidou M, Malas S, Potamitis G, et al. (2006) Novel germline mutations in the APC gene of Cypriot patients with familial and sporadic adenomatous polyposis. Clin Genet 69: 404-409.

72. Wei SC, Su YN, Tsai-Wu JJ, Wu CH, Huang YL, et al. (2004) Genetic analysis of the APC gene in Taiwanese familial adenomatous polyposis. J Biomed Sci 11: 260-265.

73. Nimura Y, Furuwatari C, Fujimori M, Fujimori Y, Nakata S, et al. (1997) Germline mutations of the APC gene in two Japanese adenomatous polyposis patients. Jpn J Hum Genet 42: 433-439.

74. Sharp A, Pichert G, Lucassen A, Eccles D (2004) RNA analysis reveals splicing mutations and loss of expression defects in MLH1 and BRCA1. Hum Mutat 24: 272.

75. Resta N, Stella A, Susca F, Montera M, Gentile M, et al. (2001) Nine novel APC mutations in Italian FAP patients. Hum Mutat 17: 434-435.

76. Dalgliesh GL, Furge K, Greenman C, Chen L, Bignell G, et al. (2010) Systematic sequencing of renal carcinoma reveals inactivation of histone modifying genes. Nature 463: 360-363.
